# Application of Commonly Used Physical Tests in a Virtual Environment in Patients With Concussion to Patients With Various Types and Severities of Acquired Brain Injury: Prospective Cohort Method Comparison Study

**DOI:** 10.2196/76995

**Published:** 2025-10-27

**Authors:** Keely Barnes, Heidi Sveistrup, Mark Bayley, Michel Rathbone, Monica Taljaard, Mary Egan, Martin Bilodeau, Motahareh Karimijashni, Shawn Marshall

**Affiliations:** 1 Acute Care Program, Ottawa Hospital Research Institute, 501 Smyth Road, Ottawa, ON, K1H 8L6, Canada, 1 613-737-8899; 2 Bruyère Health Research Institute, Ottawa, ON, Canada; 3 School of Human Kinetics Faculty of Health Sciences, University of Ottawa, Ottawa, ON, Canada; 4 Systems and Computer Engineering Technology, Carleton University, Ottawa, ON, Canada; 5 School of Rehabilitation Sciences Faculty of Health Sciences, University of Ottawa, Ottawa, ON, Canada; 6 Kite Research Institute Toronto Rehabilitation Institute, University Health Network, Toronto, ON, Canada; 7 Division of Physical Medicine and Rehabilitation, Temerty Faculty of Medicine, University of Toronto, Toronto, ON, Canada; 8 Department of Medicine, Division of Neurology, Faculty of Health Sciences, McMaster University, Hamilton, ON, Canada; 9 Methodological and Implementation Research Program, Ottawa Hospital Research Institute, Ottawa, ON, Canada; 10 School of Epidemiology and Public Health, University of Ottawa, Ottawa, ON, Canada; 11 Department of Medicine, University of Ottawa, Ottawa, ON, Canada

**Keywords:** brain injury, virtual assessment, concussion, psychometric, remote

## Abstract

**Background:**

People who sustain a concussion and live in remote areas can experience challenges in accessing specialized assessments. In these cases, virtual approaches to assessment are of value. There is limited information on important psychometric properties of physical assessment measures used to evaluate people postconcussion virtually.

**Objective:**

The aims of this method-comparison psychometric study were to determine (1) inter- and intrarater reliability of a battery of concussion physical tests administered virtually in people with brain injury and (2) sensitivity and specificity of the virtual battery when compared to the in-person assessment.

**Methods:**

A total of 60 people living with acquired brain injuries attended an in-person and virtual assessment at the Ottawa Hospital Rehabilitation Centre. The order of the assessments, in-person and virtual, was randomized. The following physical measures were administered in-person and virtually: finger-to-nose test, vestibular ocular motor screening (VOMS), static balance testing (double leg, single leg, and tandem), saccades, cervical spine range of motion, and evaluation of effort. The virtual assessment was recorded, and a second clinician viewed and independently documented findings from the recordings twice at 1-month intervals.

**Results:**

The mean age of the participants was 45.65 (SD 16.50) years. The sensitivity metrics ranged from moderate (60%, 95% CI 30-86) to excellent (100%, 95% CI 71-100) for saccades and cervical spine right lateral flexion, respectively. Specificity ranged from 75%, 95% CI 35-95 to 100%, 95% CI 91-100 for left single leg stance eyes closed and left finger-to-nose testing, respectively. The interrater reliability ranged from poor for cervical spine extension (Cohen κ=0.20, 95% CI −0.07 to 0.47) to excellent for VOMS change in symptoms (Cohen κ=0.93, 95% CI 0.83-1). The intrarater reliability ranged from poor for cervical spine extension (Cohen κ=0.31, 95% CI 0.04-0.58) to excellent for the finger-to-nose testing on the right (Cohen κ=0.90, 95% CI 0.71-1). The wide CIs highlight variability in precision and suggest that further research with larger samples is needed before clinical use can be fully standardized.

**Conclusions:**

This study provides information on the psychometric properties associated with virtual administration of concussion measures. The VOMS change in symptoms measure appears to have the most promising properties when administered virtually when in-person visits are not possible. This is particularly relevant for patients in rural areas, for those facing access barriers, and in contexts where timely follow-up is challenging. However, caution should be maintained when administering certain concussion measures virtually. The wide CIs for some measures caution against over-reliance on single test findings, and clinicians should consider both the strengths and limitations of virtual delivery. Clinicians are encouraged to make informed decisions about which measures can be effectively used remotely, and which may still require in-person administration to maintain accuracy.

## Introduction

A concussion, a form of mild traumatic brain injury, is caused by an external force to the head [1], resulting in altered brain function and commonly presenting with associated symptoms [2]. Symptom presentation varies between individuals with headaches, vision difficulties, vestibular issues, fatigue, cognitive deficits, and emotional challenges being the most common complaints [3-6]. Concussions are a public health concern impacting various groups, including athletes, workers, children, and older adults [7].

Due to the impact of concussion on multiple bodily systems, there is a need for assessments to be comprehensive in order to target the wide range of symptoms experienced [8,9]. Comprehensive assessments are typically completed by a variety of clinical professionals and are completed minutes to months post injury. The bodily systems recommended to be assessed in these examinations are typically consistent amongst clinicians and across time points [10]. Traditional comprehensive in-person assessments are considered ideal for postconcussion examinations. However, in-person assessments can be challenging for patients to attend due to geographical, resource availability, and mobility factors. Specifically, many injured individuals live in rural areas [11] and experience challenges associated with accessing nearby concussion specialists.

Virtual care alternatives offer a promising approach to assessment to increase accessibility, efficiency, and convenience [12]. In recent years, movement restrictions introduced to deal with the COVID-19 pandemic required a shift to virtual approaches to assessment for individuals with concussions [13]. This shift pushed clinicians to make clinical practice adaptations to continue to provide care for people at a distance with the aim of keeping people safe from the virus [13]. With this transition, clinicians individually determined how to adapt their assessments, with limited information on how best to do this or how effective their approaches were. Virtual assessment is continuing to be used in current practice post pandemic and has the capacity to increase the reach to patients with concussion and overcome identified barriers associated with attending in-person assessments [14]. Virtual care could offer the opportunity for more frequent touchpoints between patients and clinicians, potentially facilitating recovery. Despite this potential, there remains limited information on the psychometric properties (eg, reliability, sensitivity, and specificity metrics) associated with the virtual concussion assessment [15]. This is critical because without evidence of psychometric soundness, clinicians lack the confidence to interpret and make decisions based on virtual assessment findings. Addressing this gap is therefore necessary to establish virtual assessment as a sound extension of standard care.

We previously completed a feasibility study exploring procedures associated with virtual administration of 6 physical measures including the finger-to-nose test, vestibular ocular motor screening (VOMS) tool, balance testing (double leg stance, single leg stance, and tandem stance), saccades, cervical spine range of motion, and evaluation of effort (clinician’s subjective perception of level of effort used on assessment) [16]. We demonstrated the ability to successfully recruit participants into a study involving the completion of an in-person and virtual assessment using these measures and reported the perceived similarity associated with the 2 approaches [16]. Findings from the feasibility study informed the order of measures in the assessments, highlighted the need to explore alternate recruitment methods, and provided preliminary information on the psychometric properties. In this study, we reported results from the analysis of the full-scale study, focusing on the psychometric properties associated with virtual administration of these measures when compared to in-person administration. Our objectives were to (1) describe the interrater and intrarater reliability properties associated with the 6 physical concussion assessment measures when administered virtually in people with acquired brain injury and (2) describe diagnostic accuracy, including sensitivity and specificity of virtual administration of the 6 physical measures compared to in-person administration of the measures in people with acquired brain injury. By explicitly evaluating these properties, this study addresses a critical gap and advances the understanding of whether commonly used physical measures can be used with confidence in a virtual environment. It is important to highlight that the battery of measures assessed in this study is not reflective of a comprehensive concussion examination. We focused on commonly used physical measures intended to serve as a screening tool for select physical symptoms and signs.

## Methods

### Overview

The STROBE (Strengthening the Reporting of Observational Studies in Epidemiology) guideline for cohort studies was used to report the study. The methods are described in detail in Barnes et al [17]. We provide a brief overview below.

### Participants

#### Patient-Participants

Patient-participants included adults aged 18 years or older who had sustained a brain injury and were under the care of a clinician at the Ottawa Hospital Rehabilitation Centre. The sample included individuals with mild (concussion), moderate, and severe acquired brain injury to ensure that we included people with known abnormality on all components of the assessment. It was particularly important to capture abnormalities for finger-to-nose testing as individuals with a concussion typically perform normally on coordination testing [18,19].

#### Clinician-Participants

Clinician-participants were physiatrists, physician assistants, and physiotherapists employed at the Ottawa Hospital Rehabilitation Centre whose practice included patients with acquired brain injury.

#### Sample Size

A sample of 60 patient-participants was needed based on the estimation that the sensitivity metrics for the primary measure (VOMS) would range from 77%‐96%, and the 2-sided 95% CI around the estimated sensitivity would have a total width ranging from 8.7% to 25.3%. Further, for the reliability outcome, a sample size of 60 patient-participants provides 80% power to detect a true kappa value of 0.89 (estimated for the primary VOMS measure) using a one-sided test at the 5% significance level. The clinician-participant sample size was not predetermined. Additional information is available in Barnes et al [17].

### Recruitment and Consent

#### Patient-Participants

Purposive sampling techniques [20] were used to identify eligible participants with potential abnormalities on components of the virtual assessment. For example, we identified and recruited participants with known abnormalities on coordination testing (based on a review of the medical record) to ensure we included finger-to-nose-based deficits in our sample. This technique was used so that psychometric properties could be tabulated for each measure (ensuring variation in abnormality on each measure). Further, we purposely selected participants with varying severities of injury, varying ages and sexes, and varying mechanisms of injuries to ensure we achieved representation in our sample. Diagnoses were confirmed by the participants’ treating physiatrist, and all potential participants were reviewed by the assessing clinician to confirm suitability of participation prior to recruitment. Participants were recruited over the telephone or face-to-face through an Ontario Workers Network outpatient clinic, outpatient clinics that are publicly funded, and through inpatient rehabilitation services, all located at the Ottawa Hospital Rehabilitation Center. Patient-participants completed a form upon enrollment consisting of questions related to demographic characteristics and virtual assessment and technology experience.

#### Clinician-Participants

Clinician-participants were recruited over the telephone through the Ottawa Hospital Rehabilitation Center. Clinician-participants completed a form upon enrollment consisting of questions related to demographic and clinical practice characteristics.

### Procedures

#### Training

All recruited clinicians completed training before commencing study assessments. Training consisted of a review of a training manual developed by Johnston et al [21] and a review of the measures included in the virtual assessment. Clinicians were provided with instructions from this manual regarding how to administer the measures with slight adaptations to meet the needs of this project.

#### Assessments

REDCap (Research Electronic Data Capture; Vanderbilt University), a secure electronic data capturing system, was used to facilitate data collection and management for this study. One clinician completed a virtual and in-person assessment with the recruited patient-participants using specific physical concussion measures, including the finger-to-nose test, VOMS, saccades, static balance testing, cervical spine range of motion, and evaluation of effort. Multimedia Appendix 1 presents details of the measures administered.

The order of the assessments (in-person and virtual) was randomized. For the virtual assessment, the clinician went into a separate room from the patient-participant, within the Ottawa Hospital Rehabilitation Center, so that they could be present in case of a safety event. Microsoft Teams was used to conduct the virtual assessments, and all virtual assessments were audio-video recorded. A research team member remained in the room during the virtual assessment to ensure safety during balance and vestibular testing. The sequence of measures was identical for both the virtual and in-person assessments, with the VOMS administered last since it was most likely to aggravate symptoms, allowing clinicians to complete all other measures prior. The clinician documented their findings on a hard copy form for both assessments, which was then entered into REDCap by a research team member. Specific criteria for abnormality were used (see Barnes et al [17]) to code the findings into binary categories (normal vs abnormal). For example, if the patient-participant had a ≥2-point increase in symptoms from baseline in the VOMS, an abnormality was coded. All assessments were completed on the day of the patient-participants’ scheduled appointments, and a brief rest period was provided between each assessment. Feedback was obtained from both clinician and patient-participants after the study procedures were complete and related to perceived similarity between the 2 assessment approaches and confidence in findings obtained on the assessments.

#### Observation and Rating of Audio-Video Recordings

A clinician, different from the one who completed the initial in-person and virtual assessments, observed recordings of the virtual assessments on 2 occasions. This second clinician independently documented their findings on the virtual assessment and completed the same process approximately 1 month after initial observation.

### Analysis

The analytical approach is detailed in Barnes et al [17] Briefly, SPSS (version 28; IBM Corp) was used to calculate the sensitivity, specificity, and reliability of the virtual assessment. For sensitivity and specificity, the findings of the in-person assessment completed by the initial clinician were compared to the findings of the virtual assessment completed by the initial clinician. For interrater reliability, the virtual assessment completed by the initial clinician was compared to the independently documented findings of the second clinician. For intrarater reliability, the virtual assessment findings documented by the second clinician at 2 times, approximately 1 month apart, were compared. Cohen Kappa values were documented for reliability. We calculated 95% CIs for each statistic. As the measures administered are concussion-specific, subgroup analyses for sensitivity, specificity, and reliability were conducted for concussion only and for nonconcussion participants.

### Ethical Considerations

Ethics approval was obtained from the Ottawa Health Sciences Network Research Ethics Board (20230311‐01H), the Bruyère Health Research Ethics Board (M16-22-006), and the University of Ottawa Board of Ethics (H-06-23-9348). Informed consent was obtained from all participants before participation in the study. Privacy and confidentiality were ensured by following institutional ethical protocols. All data were deidentified prior to analyses using unique study identifiers. Recordings of the virtual assessments were stored on secure servers and were only accessible to the research team and the second clinician rater. Participants received a CAD $30 (US $22) gift card and parking voucher for taking part in the study.

## Results

### Recruitment Process

**Figure 1.**
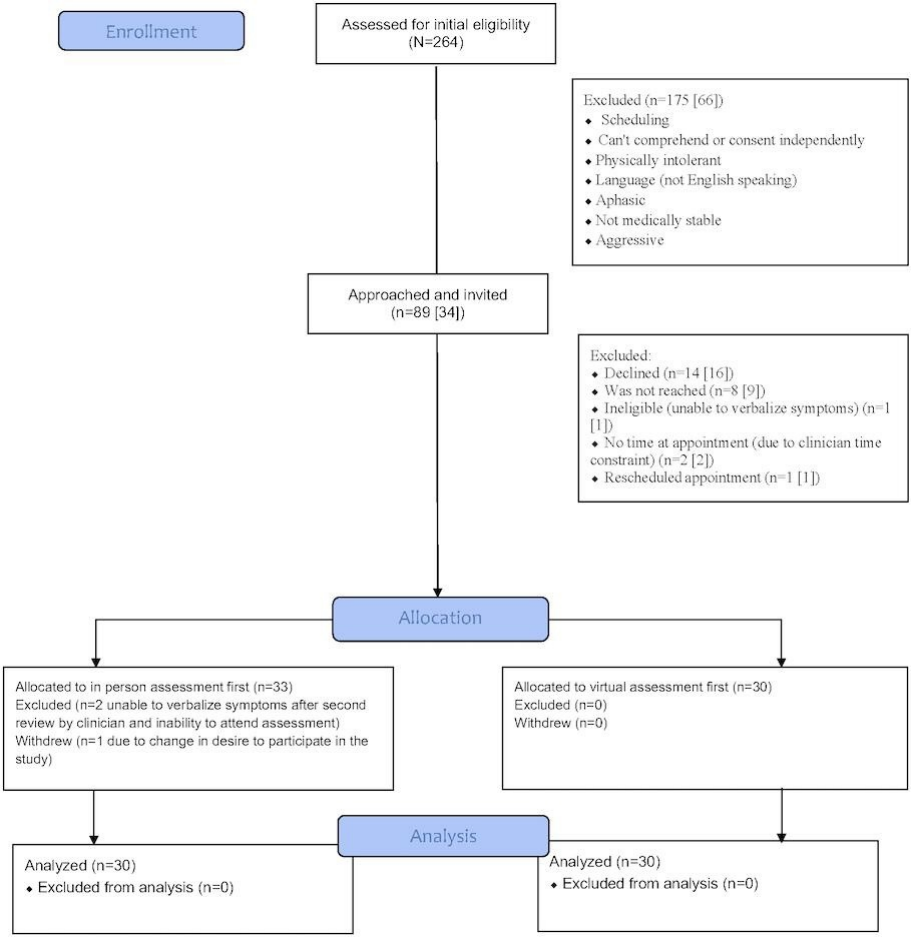
CONSORT (Consolidated Standards of Reporting Trials) diagram of participant flow in a prospective cohort method comparison study of virtual versus in-person concussion measures among adults with acquired brain injury at the Ottawa Hospital Rehabilitation Centre, July 2023-August 2024.

Figure 1 presents a CONSORT (Consolidated Standards of Reporting Trials) diagram outlining the number of participants approached and enrolled in this study.

### Demographics

#### Patient-Participant Demographic Characteristics

A total of 63 patient-participants were recruited, with 60 (95%) completing the protocol. Table 1 presents the demographic characteristics of the participants who completed the protocol. The age of the participants ranged from 18 to 78 (mean 45.65, SD 16.50) years. The majority of participants were on leave from work at the time of the study assessments, were limited in terms of their functional ability, and felt that their mental health was poor mental health. Overall, 31 of 63 (51.7%) of the participants had sustained a concussion, and the other 29 of 63 (48.3%) participants had sustained another form of brain injury (such as moderate traumatic brain injury, stroke, encephalitis, etc). The majority of brain injuries occurred in the workplace, with dates ranging from less than 6 months ago to more than 40 years ago.

**Table 1.** Demographic characteristics in a prospective cohort method comparison study of virtual versus in-person concussion measures among adults with acquired brain injury at the Ottawa Hospital Rehabilitation Centre, July 2023-August 2024.

Demographic characteristics		Values
Age (years)		
Mean (SD)		45.65 (16.50)
95% CI		45.02-46.28
Sex, n (%)		
Female		36 (60)
Male		24 (40)
Gender, n (%)	
Woman		35 (58.3)
Man		24 (40)
Gender diverse		1 (1.7)
Ethnicity, n (%)		
White		46 (76.7)
Black		5 (8.3)
Arab		1 (1.7)
Southeast Asian (eg, Vietnamese, Cambodian, Malaysian, and Laotian)		3 (5)
West Asian (eg, Iranian and Afghan)		4 (6.7)
First Nation or Indigenous		1 (1.7)
Highest educational attainment, n (%)		
Less than secondary (high) school graduation		1 (1.7)
Secondary (high) school diploma or equivalent		19 (31.7)
Some postsecondary education		3 (5)
Postsecondary certificate, diploma, or degree		37 (61.7)
Current work status, n (%)		
Off work		33 (55)
Modified return to work, same preinjury occupation		19 (31.7)
Modified return to work, different preinjury occupation		1 (1.7)
Full return to work, same preinjury occupation		4 (6.7)
Full return to work, different occupation		2 (3.3)
Other		1 (1.7)
Functional limitations, n (%)		
Moderate activities		
Yes, limited a lot		19 (31.7)
Yes, limited a little		24 (40)
No, not limited at all		17 (28.3)
Climbing stairs		
Yes, limited a lot		15 (25)
Yes, limited a little		23 (38.3)
No, not limited at all		22 (36.7)
Perceived mental health, n (%)		
Excellent or very good		10 (16.7)
Good		20 (33.3)
Fair or poor		30 (50)
Diagnosis, n (%)		
Other brain injury		29 (48.3)
Mild traumatic brain injury or concussion		31 (51.7)
Date of Injury, n (%)		
<6 months ago		22 (36.7)
6 months to <1 year ago		8 (13.3)
1 to <2 years ago		16 (26.7)
2 to <3 years ago		5 (8.3)
>3 years ago		9 (15)

Previous experience with technology and virtual assessments is reported in Table 2. Half of the participants had previously attended a virtual assessment, most participants use technology on a daily basis, and most rarely require assistance when using technology.

**Table 2.** Virtual assessment and technology experience in a prospective cohort method comparison study of virtual versus in-person concussion measures among adults with acquired brain injury at the Ottawa Hospital Rehabilitation Centre, July 2023-August 2024 (n=60).

Characteristics	Values
Previously attended virtual assessment, n (%)	
Yes	30 (50)
No	30 (50)
If yes, number attended, n (%)	
<5	21 (35)
5‐10	2 (3.3)
>10	4 (6.7)
Unsure	3 (5)
Distance living from TOHRC^a^, n (%)	
<30 minutes	30 (50)
30‐60 minutes	21 (35)
>60 minutes	8 (13.3)
Not applicable-no home	1 (1.7)
Technology available for virtual assessment, n (%)	
Computer	4 (6.7)
Laptop	16 (26.7)
Tablet	2 (3.3)
Smartphone	8 (13.3)
Multiple devices (iPad, smartphone, and computer)	28 (46.7)
None	2 (3.3)
Use of technology, n (%)	
Rarely	2 (3.3)
Monthly	3 (5)
Weekly	12 (20)
Daily	43 (71.7)
Assistance needed during the use of technology, n (%)	
Never	23 (38.3)
Rarely	22 (36.7)
Sometimes	11 (18.3)
Often	3 (5)
Always	1 (1.7)

a TOHRC: The Ottawa Hospital Rehabilitation Center.

#### Clinician-Participant Demographic Characteristics

Two physiotherapists, 2 physiatrists, and 1 physician assistant participated as clinician-assessors. All clinician-assessors had at least 5 years of clinical practice, and the volume of acquired brain injury patients seen per year was at least 50 per clinician-participant. All participants self-reported competency in completing both the in-person and virtual assessments.

#### Confidence and Perceived Similarity

Out of the total 60 patient-participants, 43 (72%) reported perceived similarity in the 2 assessment approaches, whereas 14 (25%) were unsure if similar findings were obtained or did not perceive that similar findings were obtained. Patient-participants highlighted potential disparities in measurements, eye movement tests, differences in symptoms, ability to communicate, and balance tests due to an inability of the assessor to see the whole body. The assessors reported perceived similarity for 43 (72%) of the 60 in-person and virtual assessments completed. On 14/60 (23%) occasions, clinicians did not perceive similar findings to be obtained due to patient fatigue and aggravation of symptoms, changes in balance ability, neck range, inability to observe eye movements, and the inability of the patient to follow directions as well virtually as in-person. The patient-participants were confident in their clinicians’ findings on the virtual and in-person assessments 87% (52/60) and 98% (59/60) of the time, respectively. The assessors were confident in their findings on the virtual and in-person assessment 93% (56/60) and 100% (60/60) of the time, respectively.

#### Sensitivity and Specificity

Table 3 presents the estimated sensitivity and specificity with 95% CIs (all presented as percentages) associated with virtual administration of the measures. Sensitivity ranged from 60% to 100%, representing moderate to excellent ability to identify deficits on the virtual assessment when abnormality is present on the in-person assessment. Specificity ranged from 75% to 98.2%, representing a very good to excellent ability to rule out abnormality on the virtual assessment when normality is present on the in-person assessment. The sensitivity for effort could not be calculated as there was no variation in findings.

**Table 3.** Sensitivity and specificity of the virtual assessment compared to the in-person assessment (reference standard) in a prospective cohort method comparison study of virtual versus in-person concussion measures among adults with acquired brain injury at the Ottawa Hospital Rehabilitation Centre, July 2023-August 2024.

Measures			Frequency of abnormality on in-person assessment [reference standard], n (%)	Sensitivity (95% CI)		Specificity (95% CI)	
Cervical spine ROM^a^							
Flexion			7 (11.7)	71.4^b^ (30-94)		94.3^c^ (83-99)	
Extension			19 (31.7)	73.7^b^ (49-90)		97.6^c^ (85-100)	
Right lateral flexion			13 (21.7)	100^c^ (71-100)		97.9^c^ (87-100)	
Left lateral flexion			15 (25)	93.3^c^ (66-100)		97.8^c^ (87-100)	
Right rotation			10 (16.7)	80 (44-96)		98^c^ (88-100)	
Left rotation			13 (21.7)	92.3^c^ (62-100)		97.9^c^ (87-100)	
Balance testing							
Double leg stance							
Eyes open			4 (6.7)	75^b^ (22-99)		98.2^c^ (89-100)	
Eyes closed			10 (16.7)	80^b^ (44-96)		93.9^c^ (82-98)	
Single leg stance						
Right							
Eyes open			25 (41.7)	80^b^ (60-92)		88.6^b^ (70-96)	
Eyes closed			52 (86.7)	90.4^c^ (78-96)		75^b^ (35-95)	
Left							
Eyes open			31 (51.7)	80.6^b^ (62-92)		86.2^b^ (67-95)	
Eyes closed			52 (86.7)	94.2^c^ (83-98)		75^b^ (35-95)	
Tandem stance							
Eyes open			23 (38.3)	73.9^b^ (51-89)		85.7^b^ (66-95)	
Eyes closed			35 (58.3)	85.7^b^ (69-95)		78.6^b^ (49-94)	
VOMS^d^							
Change in symptoms			35 (58.3)	91.4^c^ (76-98)		83.3^b^ (62-95)	
NPC^e^			40 (66.7)	92.5^c^ (79-98)		84.2^b^ (60-96)	
Coordination							
Finger-to-nose							
Right			12 (20)	91.7^c^ (60-100)		97.9^c^ (87-100)	
Left			12 (20)	91.7^c^ (60-100)		100^c^ (91-100)	
Oculomotor							
Saccades			10 (16.7)	60^f^ (30-86)		95.8^c^ (84-99)	
Effort							
Optimal effort			0 (0)	—^g^		100^c^ (93-100)	

a ROM: range of motion.

b Moderate: 70%-89%.

c Excellent: >90%.

d VOMS: vestibular/ocular motor screening.

e NPC: near point convergence.

f Poor: <70%.

g Not applicable.

#### Reliability

Table 4 presents the reliability properties associated with the virtual assessment. Interrater reliability metrics ranged from poor (0.20) for cervical extension to excellent (0.93) for VOMS change in symptoms. Intrarater reliability properties ranged from poor (0.31) for cervical extension to excellent (0.90) for finger-to-nose testing on the right. Effort could not be feasibly calculated as there was no variation in findings reported by clinicians.

**Table 4.** Interrater and intrarater reliability of the measures when administered virtually in a prospective cohort method comparison study of virtual versus in-person concussion measures among adults with acquired brain injury at the Ottawa Hospital Rehabilitation Centre, July 2023-August 2024.

Measures			Interrater reliability, 95% CI		Intrarater reliability, 95% CI	
Cervical spine ROM^a^						
Flexion			0.69^b^ (0.44 to 0.94)		0.48^c^ (0.19 to 0.77)	
Extension			0.20^d^ (–0.07 to 0.47)		0.31^d^ (0.04 to 0.58)	
Right lateral flexion			0.45^c^ (0.18 to 0.72)		0.55^c^ (0.29 to 0.81)	
Left lateral flexion			0.64^b^ (0.41 to 0.87)		0.70^b^ (0.48 to 0.92)	
Right rotation			0.61^b^ (0.33 to 0.89)		0.62^b^ (0.30 to 0.92)	
Left rotation			0.41^c^ (0.12 to 0.70)		0.63^b^ (0.30 to 0.96)	
Balance testing						
Double leg stance						
Eyes open			0.55^c^ (0.09 to 1)		0.79^b^ (0.40 to 1)	
Eyes closed			0.41^c^ (0.29 to 0.7)		0.52^c^ (0.24 to 0.80)	
Single leg stance						
Right						
Eyes open			0.86^b^ (0.72 to 1)		0.89^b^ (0.77 to 1)	
Eyes closed			0.71^b^ (0.45 to 0.97)		0.74^b^ (0.47 to 1)	
Left						
Eyes open			0.90^b^ (0.79 to 1)		0.76^b^ (0.59 to 0.93)	
Eyes closed			0.68^b^ (0.38 to 0.96)		0.63^b^ (0.30 to 0.96)	
Tandem stance						
Eyes open			0.92^b^ (0.81 to 1)		0.87^b^ (0.73 to 1)	
Eyes closed			0.80^b^ (0.62 to 0.98)		0.80^b^ (0.62 to 0.98)	
VOMS^e^						
Change in symptoms			0.93^b^ (0.83 to 1)		0.89^b^ (0.70 to 1)	
NPC^f^			0.44^c^ (0.20 to 0.68)		0.79^b^ (0.63 to 0.95)	
Coordination						
Finger-to-nose						
Right			0.40^d^ (0.09 to 0.71)		0.90^b^ (0.71 to 1)	
Left			0.32^d^ (0.01 to 0.63)		0.62^b^ (0.32 to 0.92)	
Oculomotor						
Saccades			0.35^d^ (0 to 0.70)		0.57^c^ (0.19 to 0.95)	

a ROM: range of motion.

b Excellent: >0.60.

c Moderate: 0.41-0.60.

d Poor: <0.40.

e VOMS: vestibular/ocular motor screening.

f NPC: near point convergence.

Results of the subgroup analyses for sensitivity, specificity, and reliability metrics for concussion and nonconcussion participants are presented in Multimedia Appendix 2.

## Discussion

### Principal Findings

We previously reported that 6 physical concussion measures, including the finger-to-nose test, VOMS, balance testing, cervical spine range of motion, and saccades, were deemed feasible and acceptable for virtual administration [16]. This study reports on the sensitivity, specificity, and reliability of the virtual administration of these measures. This is a critical advancement, as limited evidence exists on the psychometric properties of concussion-related measures in virtual environments. By directly examining reliability and diagnostic accuracy, this study provides foundational data and is essential to move beyond feasibility toward evidence-informed implementation. The findings indicate that properties vary from moderate to excellent for sensitivity and specificity, and poor to excellent for inter- and intrarater reliability.

Virtual assessments offer a potential benefit in concussion care in terms of improving accessibility, convenience, and cost-effectiveness, which is particularly important when in-person services are restricted, such as throughout the COVID-19 pandemic [12]. Highlighting these benefits may help address mixed perceptions of telehealth by demonstrating the tangible value that virtual assessments can add to both patients and health systems. While the benefits of virtual assessments are clear, documenting the reliability, sensitivity, and specificity metrics associated with the virtual administration of measures is crucial because clinicians rely on findings obtained on these assessments to make important clinical decisions, such as the need for directed treatment. Without this evidence, adoption of virtual care risks being undermined by uncertainty about accuracy. The findings of this study provide some insight into the important properties and identify the measures that offer the most promise when administering virtually. This, in turn, may inform clinical practice, ensuring that quality of care is maintained in a virtual environment.

The psychometric properties for in-person assessment of the 6 measures we tested have been reported previously and vary between studies. The sensitivity metrics associated with in-person administration range from poor (0.45 for the single-leg stance test) [22] to excellent (0.96 for the VOMS) [23]. Adequate sensitivity properties are important to ensure that the presence and magnitude of the deficits are appropriately identified by clinicians, which in turn aids in informing management [24]. The reliability of in-person administration of the measures ranges from moderate (κ=0.54 for finger to nose testing) [25] to excellent (intraclass correlation coefficient=0.90 for cervical spine range of motion evaluation) [26]. Acceptable reliability properties are required to ensure that measures yield consistent results required to make informed clinical decisions.

While acknowledging the variability in properties for in-person administration of measures, the findings of this study indicate that certain measures may be more suitable to administer virtually compared to others. The reliability, sensitivity, and specificity properties range from poor to excellent for all measures when administered virtually, with the VOMS change in symptoms measure showing the most promising metrics. Interrater reliability properties appear to be poor for most cervical spine range of motion evaluations, simpler balance tests (double leg stance, eyes open and closed), finger-to-nose testing, and oculomotor tests, including saccades and near point convergence measurement. These objective findings are in line with subjective concerns regarding the reliability and accuracy associated with the virtual assessment and the ability to identify subtle deficits over videoconferencing [27]. This highlights a potential area for development through the exploration of technological advancements to support completion of the virtual assessments, with a needed focus on ocular, simple balance, and coordination measures [28].

Reliability properties associated with the virtual administration of measures previously reported in the literature appear to be superior compared to those obtained in this study. Measures such as the 30-second arm curl test, 30-second chair stand test, 2-minute step test [29], knee and wrist joint range of motion [30], Berg Balance Scale, Timed Up and Go, Dynamic Gait Index [31], and the Tinetti Performance-Oriented Mobility Assessment gait scale [32] contain good to excellent reliability properties when administered in a virtual environment. Potential explanations for the variations in reliability metrics could include differences in technology and equipment used, differences in patient populations (with people with concussion potentially experiencing more subtle deficits that may be more challenging to identify on videoconferencing platforms), differences in measures used (with the concussion measures relying more on subjective interpretation by clinicians), and differences in methodological approaches (relying on recordings of videos compared to administering twice in the virtual environment).

When considering sensitivity and comparing the subjective perceptions of the participants, the perceived similarity appears to be comparable to the objective findings on the assessments, with 75% of participants perceiving as though similar results were obtained and sensitivity metrics above 75% for the majority of measures. This is superior to metrics reported in the literature for measures such as extraocular movements, gait, sensation, facial weakness [33], reach [34], and the Nine Hole Peg Insertion Test [35]. Superior sensitivity metrics reported in this study may be due to methodological differences (comparing the findings of the same clinician vs different clinicians), and clinician familiarity with the participants.

While there are clear concerns regarding the similarity of virtual and in-person administration of certain measures, previous studies have documented strong associations between in-person and virtual administration of stroke and multiple sclerosis measures [36-38].

In terms of participant perceptions of the assessments, a high level of confidence and perceived similarity was documented in this study. This is consistent with findings documented by Robb et al [39] who compared telemedicine and in-person visits, consisting of a clinical history interview and focused neurological examination (including gait, ocular movements, pronator drift, finger-to-nose, finger tapping, facial motor symmetry, and brief mental status evaluation) completed by a neurologist, for people with multiple sclerosis. Robb et al [39] reported perceived equivalence on the 2 approaches and highlighted the value in offering virtual visits as an alternative to in-person care. From the neurologist’s perspective, the virtual approach provided similar information when compared to the in-person visit.

While an understanding of psychometric properties associated with clinical measures is crucial, the selection of measures to use in virtual practice by clinicians also depends on the clinical utility characteristics. Previous work has reported that clinical instinct is prioritized over the use of standardized measures [40]. Physiotherapists may use standardized measures to quantify ability; however, clinical decisions regarding management typically rely upon observation [41]. Clinical relevance, as perceived by clinicians, acts as a facilitator to the use of certain measures in practice, and therefore, clinical utility may be of more importance to the use of measures rather than solely relying on empirical data [42]. Given these insights, clear communication of the properties associated with virtual administration of measures obtained in this study, along with emphasis on clinical relevance and ease of use for certain measures (such as the VOMS) compared to others (such as cervical spine range of motion), will be needed. It is recommended that clinicians exercise caution when using certain clinical measures virtually due to the variable psychometric properties. However, the integration of virtual assessments (using measures with promising properties) as a complement to in-person assessments may enhance the capacity to support patients through recovery. Further, there is potential for technological advancements, such as wearable sensors, to improve the accuracy of certain assessments. Development of a virtually appropriate battery of tests is needed. It should also be noted that several of the estimates in this study had wide confidence intervals reflecting variability in precision. These wide intervals highlight the need for cautious interpretation and reinforce the importance of future studies with larger sample sizes to narrow confidence intervals and strengthen clinical recommendations.

### Limitations

To address potential sources of bias, several standardization procedures were implemented. All study assessments were completed at the Ottawa Hospital Rehabilitation Center to ensure consistency in testing environment and equipment. The order of virtual and in-person assessments was randomized and counterbalanced to reduce rater and order effects. In addition, the virtual assessments were recorded and independently rated on 2 occasions by a second clinician to minimize single-rater bias and enable evaluation of both inter- and intrarater reliability.

However, certain metrics, such as sensitivity and specificity, may be overestimated as both the in-person and virtual assessments were completed in the same setting and with clinicians who were familiar with the patients. This limits the generalizability of the findings, as factors present in home environments with variable access to technology may impact psychometric properties associated with the measures. Technical challenges may contribute to more difficulties associated with completing virtual assessments in home environments [43]. Therefore, properties may differ if virtual assessments were conducted in a true virtual environment, such as in the home setting.

Sample bias may be present, as participants comfortable with technology may have been more inclined to participate when compared to those with limited experience or comfort with technology. Furthermore, rater bias may have contributed to properties observed between the in-person and virtual assessments; however, in attempts to address this issue, the order of the assessments was randomized and counterbalanced. In evaluating reliability, additional biases may be present due to duplicative testing in the in-person and virtual environments. We report on the top 6 physical concussion measures as identified by expert clinicians [27,44] and 4 of the most critical psychometric properties; however, future work should expand on the measures explored in the virtual environment and investigate additional properties such as responsiveness [45].

Some of the measures used in this study may not be relevant or validated in specific forms of acquired brain injury, such as the VOMS, which has not been explored for use in populations other than concussion. Therefore, properties for use in the nonconcussion population remain uncertain. Subgroup analyses (Multimedia Appendix 2), however, potentially support generalization beyond concussion for certain measures.

### Conclusions

Clinical measures with acceptable psychometric properties are required for widespread adoption of such measures in practice. This method-comparison study reports on the reliability, sensitivity, and specificity associated with the virtual administration of certain physical concussion measures, including the finger-to-nose test, cervical spine range of motion, balance testing, VOMS, and saccades, along with the evaluation of effort. Metrics associated with these measures vary from poor to excellent. The virtual approach to concussion physical assessment may provide a promising approach to complement in-person care when barriers to attending face-to-face appointments exist. However, it is recommended that clinicians consider properties when interpreting certain measures, such as the VOMS and complex balance tests. Further research is needed to expand on these findings to include the exploration of other measures, additional psychometric properties, and the potential for technology to improve the ability to accurately and consistently identify deficits post concussion.

## Supplementary material

10.2196/76995 Multimedia Appendix 1 Overview of measures administered in the assessments.

10.2196/76995 Multimedia Appendix 2 Subgroup analyses.

10.2196/76995 Checklist 1 STROBE Checklist.

## Abbreviations

CONSORT: Consolidated Standards of Reporting Trials
REDCap: Research Electronic Data Capture
STROBE: Strengthening the Reporting of Observational Studies in Epidemiology
VOMS: vestibular/ocular motor screening
