# Supplementary material for: Application of Commonly Used Physical Tests in a Virtual Environment in Patients With Concussion to Patients With Various Types and Severities of Acquired Brain Injury: Prospective Cohort Method Comparison Study
Source: J Med Internet Res. 2025 Oct 27;27:e76995. doi: 10.2196/76995 (PMC12558424; doi:10.2196/76995)
Supplement: Multimedia Appendix 1 [file jmir-v27-e76995-s001.docx]

**Table S1.**

| **Measures** | **Description for the identification of abnormality** | **Adaptations for virtual administration** |
| --- | --- | --- |
| Finger-to-nose | Hesitation, tremor, under or overshooting | Touches own finger instead of examiners |
| VOMS | Change in symptoms greater than or equal to two points out of 10  Near point convergence component of the VOMS: distance greater than or equal to five centimeters | Uses own fingers instead of examiners  Looks at examiners nose on screen for VOR instead of examiners finger |
| Balance (feet together, single leg stance, tandem stance) eyes open and eyes closed for 20 seconds each | Inability to hold the position for 20 seconds | Stands in a corner or close to supports |
| Saccades | Speed, accuracy, initiation, intrusions or oscillations, range of motion and conjugacy | Uses own fingers instead of examiners |
| Range of motion (flexion, extension, right and left lateral flexion, right and left rotation) | Values compared to pooled norms: flexion = 50-72°; extension = 58-77°; lateral flexion = 37-47°; rotation = 67-81° | No use of device for measurements |
| Effort rating scale | Documentation of perceived effort on a scale of 0 (no effort) to 10 (full effort) | NA |

**NA**, not applicable; **ROM**, range of motion; **VOMS**, Vestibular/Ocular Motor Screening; **VOR**, Vestibulo-ocular reflex
