# Supplementary material for: Application of Commonly Used Physical Tests in a Virtual Environment in Patients With Concussion to Patients With Various Types and Severities of Acquired Brain Injury: Prospective Cohort Method Comparison Study
Source: J Med Internet Res. 2025 Oct 27;27:e76995. doi: 10.2196/76995 (PMC12558424; doi:10.2196/76995)
Supplement: Multimedia Appendix 2 [file jmir-v27-e76995-s002.docx]

**Table S1.** Subgroup sensitivity and specificity analyses for concussion-only versus nonconcussion participants.

|  | | | **Concussion-Only Participants**  **(N=31)** | | **Non-Concussion Participants**  **(N=29)** | |
| --- | --- | --- | --- | --- | --- | --- |
| **Measure** | | | **Sensitivity (%)** | **Specificity (%)** | **Sensitivity (%)** | **Specificity (%)** |
| **Cervical Spine ROM** | | | | | | |
| Flexion | | | 75 | 92.6 | 66.7 | 96.2 |
| Extension | | | 80 | 100 | 66.7 | 95 |
| Right lateral flexion | | | 100 | 96 | 100 | 100 |
| Left lateral flexion | | | 85.7 | 100 | 100 | 95.2 |
| Right rotation | | | 75 | 96.3 | 83.3 | 100 |
| Left rotation | | | 100 | 96 | 85.7 | 100 |
| **Balance Testing** | | | | | | |
| Double leg stance | Eyes open | | 100 | 96.7 | 66.7 | 100 |
|  | Eyes closed | | 80 | 88 | 80 | 100 |
| Single leg stance | Right | Eyes open | 85.7 | 87.5 | 77.8 | 90.9 |
|  |  | Eyes closed | 87 | 75 | 93.1 | - |
|  | Left | Eyes open | 77.8 | 86.4 | 81.8 | 85.7 |
|  |  | Eyes closed | 91.3 | 75 | 96.6 | - |
| Tandem stance | Eyes open | | 44.4 | 85.7 | 92.9 | 85.7 |
|  | Eyes closed | | 84.6 | 87.5 | 86.4 | 66.7 |
| **VOMS** | | | | | | |
| Change in symptoms | | | 92 | 60 | 90 | 89.5 |
| NPC | | | 100 | 77.8 | 84.2 | 90 |
| **Coordination** | | | | | | |
| Finger-to-nose | Right | | 50 | 96.6 | 100 | 100 |
|  | Left | | 50 | 100 | 100 | 100 |
| **Oculomotor** | | | | | | |
| Saccades | | | 50 | 96 | 66.7 | 95.7 |
| **Effort** | | | | | | |
| Optimal effort | | | - | 100 | - | 100 |

**NPC**, near point convergence; **ROM**, range of motion; **VOMS**, Vestibular/Ocular Motor Screening

Table S2. Subgroup reliability analyses for concussion-only versus nonconcussion participants.

|  | | | **Concussion-Only Participants**  **(N=31)** | | **Non-Concussion Participants**  **(N=29)** | |
| --- | --- | --- | --- | --- | --- | --- |
| **Measure** | | | **Interrater reliability** | **Intrarater reliability** | **Interrater reliability** | **Intrarater reliability** |
| **Cervical Spine ROM** | | | | | | |
| Flexion | | | 0.67 | 0.58 | 0.7 | 0.34 |
| Extension | | | 0.15 | 0.2 | 0.25 | 0.43 |
| Right lateral flexion | | | 0.38 | 0.45 | 0.49 | 0.59 |
| Left lateral flexion | | | 0.53 | 0.6 | 0.7 | 0.77 |
| Right rotation | | | 0.52 | 0.78 | 0.66 | 0.52 |
| Left rotation | | | 0.31 | 0.35 | 0.52 | 0.84 |
| **Balance Testing** | | | | | | |
| Double leg stance | Eyes open | | - | - | 0.78 | 0.78 |
|  | Eyes closed | | 0.23 | 0.27 | 0.59 | 0.63 |
| Single leg stance | Right | 0.74 | 0.79 | 0.93 | 0.93 | 0.93 |
|  |  | 0.71 | 0.76 | 0.65 | 0.65 | 0.65 |
|  | Left | 0.85 | 0.59 | 0.92 | 0.92 | 0.85 |
|  |  | 0.67 | 0.76 | 0.65 | 0.65 | - |
| Tandem stance | Eyes open | | 0.77 | 0.75 | 1.0 | 0.93 |
|  | Eyes closed | | 0.72 | 0.82 | 0.89 | 0.76 |
| **VOMS** | | | | | | |
| Change in symptoms | | | 1.0 | 1.0 | 0.86 | 0.79 |
| NPC | | | 0.38 | 0.68 | 0.44 | 0.86 |
| **Coordination** | | | | |  |  |
| Finger-to-nose | Right | | 0.65 | 1.0 | 0.29 | 0.87 |
|  | Left | | 0.65 | 0.65 | 0.2 | 0.58 |
| **Oculomotor** | | | | | | |
| Saccades | | | 0.63 | 0.47 | 0.14 | 0.63 |
| **Effort** | | | | | | |
| Optimal effort | | | - | - | - | - |

**NPC**, near point convergence; **ROM**, range of motion; **VOMS**, Vestibular/Ocular Motor Screening
